# Association of Genetic Variants in Wnt Signaling Pathway with Tuberculosis in Chinese Han Population

**DOI:** 10.1371/journal.pone.0093841

**Published:** 2014-04-02

**Authors:** Xuejiao Hu, Mengqiao Shang, Juan Zhou, Yuanxin Ye, Xiaojun Lu, Chuanmin Tao, Binwu Ying, Lanlan Wang

**Affiliations:** Department of Laboratory Medicine, West China Hospital, Sichuan University, Chengdu, Sichuan Province, People's Republic of China; University of Minnesota, United States of America

## Abstract

Compelling studies have implicated that the Wnt signaling pathway plays an important role in the development and progression of tuberculosis, however, there is little literature addressing the role of polymorphisms in Wnt pathway on tuberculosis. We took a pathway based candidate gene approach to investigate the possible correlation between genetic variants in Wnt pathway and tuberculosis. Three single nucleotide polymorphisms (SNPs) in Wnt pathway (rs4135385 in CTNNB1 gene, rs7832767 in SFRP1 gene, and rs11079571 in AXIN2 gene) were genotyped in 422 Chinese Han tuberculosis patients and 402 frequency matched (age, gender, and ethnicity) controls using high-resolution melting analysis. The genotype and allelic frequencies of rs4135385 and rs7832767 were significantly different among patients and controls. The dominant model of rs4135385 was significantly associated with an increased risk of tuberculosis (AG/GG versus AA: OR = 1.49, 95% CI = 1.06–2.09, *p* = 0.019). The recessive model of rs7832767 posed a significant higher risk for tuberculosis (TT versus TC/CC, OR = 2.70, 95% CI = 1.41–5.18, *p* = 0.002). These SNPs were further evaluated whether they were correlated with the site of tuberculosis and the level of inflammatory markers. Rs7832767 was significantly associated with the level of CRP (*p* = 0.014), and the patients carrying T allele might present with elevated CRP values (OR = 1.90, 95% CI = 1.21–2.96, *p* = 0.005). Our study provided the first evidence that rs4135385 and rs7832767 were associated with tuberculosis risk, and genetic variants in Wnt signaling pathway might participate in genetic susceptibility to tuberculosis in Chinese Han population. Further epidemiological and functional studies in larger populations are warranted to verify our results.

## Introduction

Tuberculosis (TB) remains a leading cause of morbidity and mortality caused by infectious agents throughout the world, especially in developing countries like China. Epidemiological data have shown that only 10% of *Mycobacterium tuberculosis* (MTB) infection will finally develop into the clinically active disease [1]. The progression to active TB depends on the interplay between environmental elements, host genetic factors and pathogenic characteristics of MTB [2]. Among them, host susceptibility to MTB has evoked concern of numerous scholars recently. Abundant studies have investigated genetic variants mainly involved in innate and adaptive immunity to TB [3]–[5], and molecular pathways that regulate immune response, for instance, JAK/STAT [6], MAPK [7], NF-κB signaling [8], have been a hot area for TB infection research. Some gene factors within signaling pathways may be molecular targets for MTB infection, which provide novel insights into the pathogenesis of tuberculosis.

The Wnt signaling pathway, an ancient master signaling network governing ontogeny and homeostatic processes, has recently been demonstrated to effect immune response in a variety of inflammatory and infectious diseases [9]–[11]. The canonical Wnt signaling pathway is firstly activated when Wnt molecules bind to LRP5/6-Frizzled receptors, and then AXIN complex phosphorylate and sequestrate in the cytoplasm. As a result, a number of β-catenin molecules in the cytoplasm accumulate and translocate into the nucleus where they control the transcription of some target genes. Besides, the secreted frizzled related protein (SFRP) family (SFRP1–5) inhibit the Wnt pathway by binding to Wnt proteins and prevent their interactions with Frizzleds, acting as negative regulators of canonical signaling activation and target gene expression [9], [12]. Compelling evidences have implicated that the Wnt pathway involves in the pathogenesis of TB [11]–[14]. Blumenthal et al. [14] demonstrated that both WNT5A and Frizzled-5 regulated critical antimicrobial effector functions in response to MTB. Neumann et al. [12] found most of the Frizzled family and AXIN2 were significantly down-regulated in MTB–infected mice. Schaale K et al. [11] observed that the majority of Wnt homologs mRNA expression were significantly reduced during MTB infection, and further demonstrated that Wnt6 was expressed in granulomatous lesions and played an important role in macrophage differentiation and proliferation. The role of Wnt signaling pathway in TB has recently been payed attention but not fully understood yet. To date, there are little data assessing epidemiologic genetic association studies of Wnt signaling pathway with tuberculosis. In order to investigate potential association between genetic polymorphisms in the Wnt pathway with TB risk, we genotyped three single nucleotide polymorphisms (SNPs) from key genes within Wnt signaling pathway (i.e. rs4135385 in CTNNB1 gene, rs7832767 in SFRP1 gene, rs11079571 in AXIN2 gene) in a case-control study (422 tuberculosis patients and 402 healthy controls) in Chinese Han population.

## Materials and Methods

### Subjects

A total of 422 TB patients and 402 healthy controls were enrolled in the present study. Patients with newly diagnosed TB were consecutively recruited from West China Hospital of Sichuan University, from April 2011 to October 2013. All patients satisfied the inclusion criteria for established TB: smear positive for at least two separate specimens and/or culture positive for MTB and/or clinical and radiological findings consistent with TB and/or pathological evidence of TB disease. They never received anti-tuberculosis treatments, or medication durations less than half of a month. Patients with HIV-seropositive result, other infectious diseases or immunosuppression were excluded. The 402 controls was randomly selected from a pool of more than 2,500 tuberculosis-free individuals on the basis of normal radiographic findings and physical examinations, tuberculin skin test results <5 mm, no history of TB, and frequency matched to the cases on age and sex. All subjects were from the same district and genetically unrelated ethnic Han Chinese. This study was approved by the ethical committee of West China Hospital, Sichuan University, and signed informed consent forms were obtained from all the subjects.

### Gene and SNP selection

Candidate genes were selected based on their functional significance as key components of the Wnt signaling pathway. Candidate SNPs were identified via searching the International HapMap Project (http://www.hapmap.org/index.html.en), and dbSNP database (http://www.ncbi.nlm.nih.gov/projects/SNP/). SNPs with minor allele frequency (MAF) >0.15 in Chinese Han population were considered further. Combined with previous literature [15]–[17], we finally included three SNPs from three key genes within Wnt signaling pathway (rs4135385 A>G in CTNNB1, rs7832767 C>T in SFRP1, rs11079571 A>G in AXIN2 gene), as shown in Table 1.

**Table 1:** Primer sequences for the three SNPs genotyping.

SNP	SNP ID	Location	Allele	Primer sequence (from 5′- to 3′-end) F: forward R: reverse	Size (bp)
CTNNB1	rs4135385	intron	A/G	F:5′-GCATTTGTGTAATGTTGGAGTTAC-3′	120
				R:5′-CATGCACAAAGCAAGGAAGAAT-3′	
SFRP 1	rs7832767	intron	C/T	F:5′-TACGCGCTCTCCAGTTTAGC-3′	157
				R:5′-CCTGCCCTGTCAAAGTAAGC-3′	
AXIN2	rs11079571	intron	A/G	F:5′-GTGATGAGGCCACATTCTGT-3′	150
				R:5′-ATGATCCAACTGCCTGCTCT-3′	

### Genotyping analysis

Three milliliter EDTA-anticoagulated peripheral blood samples were obtained from all participants. Genomic DNA was extracted using QIAamp® DNA Blood mini kit (Qiagen, Germany) according to the manufacturer's protocol, and finally the DNA diluted to 20 ng/μL for experiment. SNP genotyping was performed using high-resolution melting (HRM) analysis, based on a melting temperature shift or an altered shape of the melting curve of the PCR products. A volume of 20 ul PCR reaction mixture contained 20 ng genomic DNA, 5 pmol of forward and reverse primers, 62.5 nmol Mg^2+^ and 1×Roche HRM mixture (Roche Diagnostics, Germany). PCR amplification was carried out in a 96-well plate in the LightCycler® 480 Real-Time PCR System (Roche Diagnostics, Germany). The thermal cycling profile was initial denaturation at 95 °C for 15 min, then amplification for 50 cycles by denaturing at 95 °C for 10 s, annealing at 60 °C for 15 s, and extension at 72 °C for 25 s. After amplification, PCR products were denatured at 95 °C for 1 min and cooled to 40 °C for 1 min to form double-strand DNA. Then the HRM analyses were performed by gradually increasing the temperature from 65 °C to 95 °C at a rate of 0.01 °C/s. Obtained data were analyzed using Gene Scanning software v1.2 (Roche Diagnostics, Germany). The clearly distinguished genotype melting curves were finally included. Double distilled water (PCR grade) as a negative control was used to ensure the accuracy of PCR process. Five percent of all samples were randomly selected and genotyped by both HRM and sequencing. Samples with three different genotypes based on the sequence results (such as genotypes AA, AG, GG) were selected as the references. According to the referent genotypes, the genotypes of samples were defined.

### C-reactive protein (CRP) and erythrocyte sediment rate (ESR) detection

The blood specimens were from all the patients on admission. CRP was measured by rate nephelometric assay with the fully automated analyzer IMMAGE® 800 (Beckman Coulter, USA). Serum CRP level >5 mg/L was elevated. ESR was detected with EDTA-anticoagulated peripheral blood in automatic ESR analyzer Test 1 (ALIFAX, Italy), according to the manufacturer's instructions. The reference range of ESR defined 0–15 mm/h for male, 0–20 mm/h for female.

### Statistical analysis

All genotype frequencies were analyzed for Hardy–Weinberg equilibrium using the goodness-of-fit χ^2^ test. Differences in allele/genotype frequencies between groups were obtained using Chi-square (χ^2^) test. Odds ratio (OR) and 95% confidence intervals (CI) were calculated to assess the relative risk conferred by a certain allele or genotype. All statistical analyses were performed with SPSS software (version 16.0, SPSS Inc., USA), and statistical significance was established at an alpha level of 0.05.

## Results

### Basic characteristics of subjects

The study cohort was composed of 422 TB patients (248 males [58.77%] and 174 females [41.23%], mean age [interquartile range, IQR] = 42.7 [32.7–54.2] years) and 402 control subjects (240 males [59.70%] and 162 females [40.29%], mean age [IQR] = 43.2 [30.7–55.8] years) from Chinese Han population. No significant differences in age and sex were observed among patients and controls (*p*>0.05).

### Association of SNPs with TB in Wnt signaling pathway

**Figure 1:**
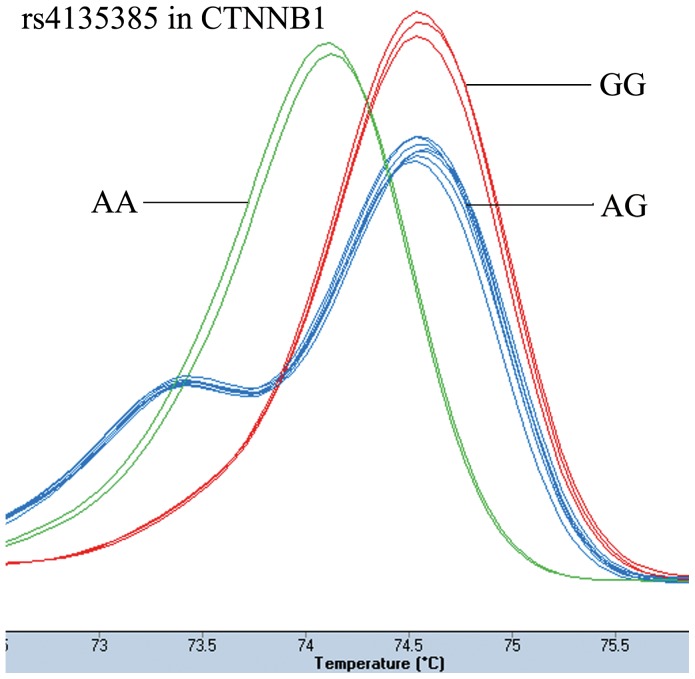
HRM plot of rs4135385.

**Figure 2:**
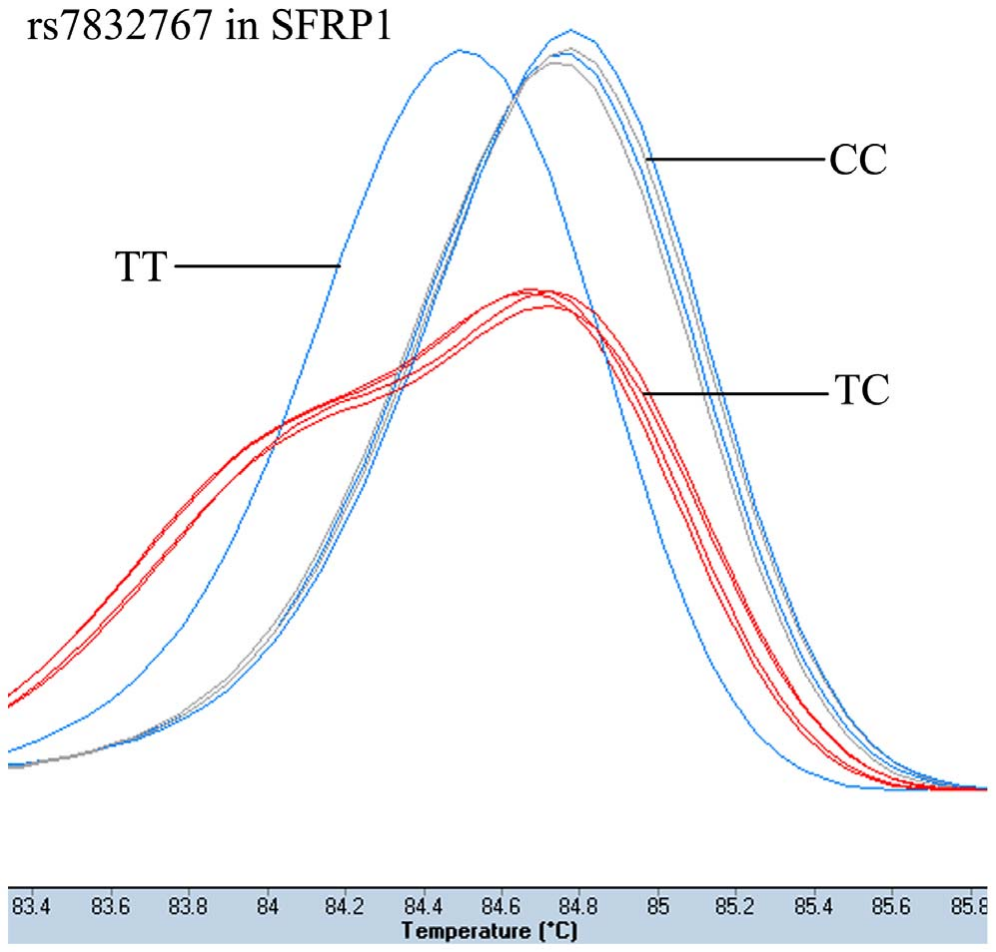
HRM plot of rs7832767.

**Figure 3:**
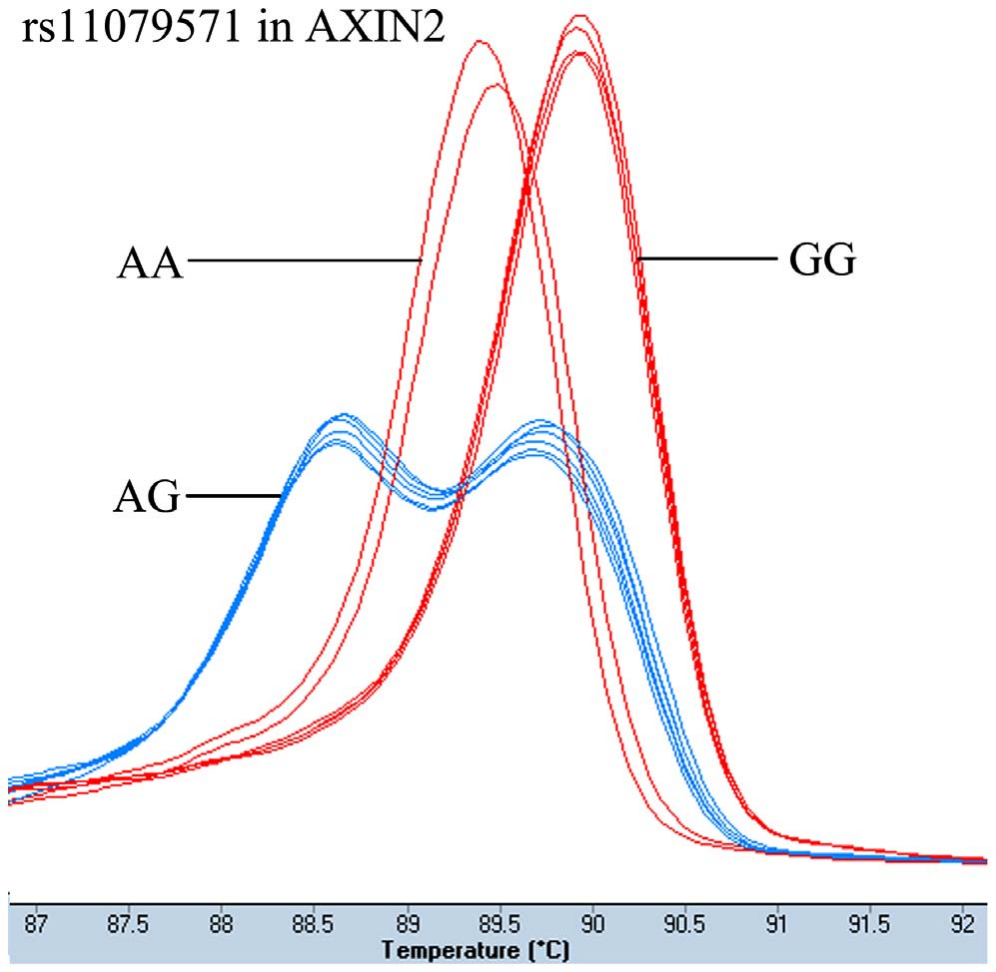
HRM plot of rs11079571.

The patient group had been genotyped successfully 421 cases for rs4135385 and rs11079571, 422 cases for rs7832767. The number of the controls with clear genotype results was: 400 cases for rs4135385, 401 cases for rs7832767, and 402 cases for rs11079571. The genotype results of HRM analysis and sequencing were 100% concordance. Figure 1–3 showed the HRM plots for three SNPs.

Genotype distributions of three SNPs in cases and controls were all in agreement with Hardy-Weinberg equilibrium (*p*>0.05). Table 2 displayed the genotype and allelic frequencies of three SNPs. The distributions of genotype and allelic frequencies of rs4135385 were significantly different among cases and controls (*p* = 0.048, 0.020, respectively), and G allele might be a risk factor for TB (OR = 1.26, 95% CI: 1.04–1.53, *p* = 0.020). There were significant differences in both genotype and allelic distributions of rs7832767 among patients and controls (*p* = 0.008, 0.047, respectively), and subjects carrying T allele exhibited an increased tuberculosis risk (OR = 1.26, 95% CI: 1.01–1.58, *p* = 0.047). No statistical significances were observed with regard to the genotype and allelic frequencies of rs11079571 between two groups (*p*>0.05).

**Table 2:** Comparison of Wnt pathway gene polymorphisms between TB patients and controls.

SNP		Case n(%)	Control n(%)	OR(95%CI)	*P*		Case n(%)	Control n(%)	*P*
rs4135385	G	484(57.48)	414(51.75)	**1.26(1.04–1.53)**	**0.020**	AA	75(17.81)	98(24.50)	**0.048**
	A	358(42.52)	386(48.25)			AG	208(49.41)	190(47.50)	
						GG	138(32.78)	112(28.00)	
rs7832767	T	217(25.71)	173(21.57)	**1.26(1.01–1.58)**	**0.047**	TT	35(8.29)	13(3.24)	**0.008**
	C	627(74.29)	629(78.43)			TC	147(34.83)	147(36.66)	
						CC	240(56.88)	241(60.10)	
rs11079571	G	555(65.91)	549(68.28)	1.11(0.91–1.37)	0.307	AA	50(11.90)	42(10.45)	0.595
	A	287(34.09)	255(31.72)			AG	187(44.40)	171(42.54)	
						GG	184(43.70)	189(47.01)	

We further analyzed two different inheritance models including dominant model (variant-containing genotypes versus homozygous wild-type genotype) and recessive model (homozygous variant genotype versus wild-type-containing genotype) (Table 3). The better fitting model was the one with a smaller *p* value. In the dominant model, the subjects carrying AG/GG genotype in rs4135385 showed a 1.49-fold increased risk of TB (AG/GG versus AA: OR = 1.49, 95% CI = 1.06–2.09, *p* = 0.019). In the recessive model of rs7832767, the TT genotype posed a 2.70-fold higher risk for TB (OR = 2.70, 95% CI = 1.41–5.18, *p* = 0.002) when compared with the TC/CC genotype. The results of inheritance mode suggest AG/GG genotype of rs4135385 and TT genotype of rs7832767 might be risk factors for TB.

**Table 3:** Association of Wnt pathway gene polymorphisms with TB risk.

SNP	Genotype	Case/Control	?^2^	OR(95%CI)	*P*
rs4135385	AG+GG	346/302	5.511	**1.49(1.06–2.09)**	**0.019**
	AA	75/98			
rs7832767	TT	35/13	9.555	**2.70(1.41–5.18)**	**0.002**
	TC+CC	387/388			
rs11079571	GG	184/189	0.909	0.88(0.67–1.15)	0.340
	AA+AG	237/213			

### SNPs and the location of tuberculosis

In order to identify whether the polymorphisms of Wnt pathway associated with the site of tuberculosis, we divided the patients into 162 pulmonary tuberculosis (PTB) patients and 260 pulmonary combined extra-pulmonary tuberculosis (PTB combined EPTB) patients. PTB combined EPTB refers to TB infection that infects other body organs as well as lung. There were no links between three SNPs and the location of tuberculosis (Table 4).

**Table 4:** Comparison of SNPs in PTB versus PTB combined extra-PTB patients.

SNP		P/P&E*	OR(95%CI)	*P*		P/P&E	*P*
rs4135385	G	185/299	1.03(0.78–1.36)	0.859	AA	29/46	0.972
	A	139/219			AG	81/127	
					GG	52/86	
rs7832767	T	84/133	1.02(0.74–1.40)	0.910	TT	18/17	0.090
	C	240/387			TC	48/99	
					CC	96/144	
rs11079571	G	204/351	1.24(0.92–1.65)	0.153	AA	22/28	0.354
	A	120/167			AG	76/111	
					GG	64/120	

* Pulmonary tuberculosis/Pulmonary combined extra-pulmonary tuberculosis.

### SNPs and inflammatory makers

CRP and ESR are common inflammatory markers in most laboratories. It has been found that level of these two markers have great value in evaluating the activity and severity of TB in a certain extent [18]–[19]. The patients were stratified by the level of CRP and ESR to further investigate possible relationships between SNPs in Wnt pathway and inflammatory makers. As shown in Table 5, rs7832767 showed a significant difference between high CRP group and normal CRP group (*p* = 0.014), and the subjects carrying T allele may present with elevated CRP values (OR = 1.90, 95% CI = 1.21–2.96, *p* = 0.005). There were no statistical differences in polymorphisms of three loci between different ESR subgroups (*p*>0.05) (Table 6).

**Table 5:** Comparison of SNPs in high CRP group versus normal CRP group.

SNP		H/N*	OR(95%CI)	*P*		H/N	*P*
rs4135385	G	395/89	1.10(0.78–1.55)	0.598	AA	59/1	0.842
	A	287/71			AG	169/39	
					GG	113/25	
rs7832767	T	190/27	**1.90(1.21–2.96)**	**0.005**	TT	32/3	**0.014**
	C	494/133			TC	126/21	
					CC	184/56	
rs11079571	G	454/101	1.16(0.81–1.67)	0.408	AA	37/13	0.380
	A	228/59			AG	154/33	
					GG	150/34	

* High CRP group/Normal CRP group.

**Table 6:** Comparison of SNPs in high ESR group versus normal ESR group.

SNP		H/N*	OR(95%CI)	*P*		H/N	*P*
rs4135385	G	422/62	1.37(0.93–2.01)	0.107	AA	59/16	1.171
	A	298/60			AG	180/28	
					GG	121/17	
rs732767	T	180/37	1.31(0.86–2.00)	0.207	TT	28/7	0.473
	C	542/85			TC	124/23	
					CC	209/31	
rs11079571	G	470/85	1.22(0.81–1.85)	0.344	AA	46/4	0.397
	A	250/37			AG	158/29	
					GG	156/28	

* High ESR group/Normal ESR group.

## Discussion

Understanding genetic factors that influence host susceptibility could provide novel perspectives for pathophysiological characteristics of TB, and it also might be valuable to facilitate individualized treatment decisions [20]. For the first time, we took a pathway based candidate gene approach to investigate TB risk association with three SNPs in the Wnt signaling pathway in Chinese Han population. The results indicated that rs4135385 in CTNNB1, rs7832767 in SFRP1 gene were correlated with TB susceptibility, and rs7832767 was found to be associated with the expression level of CRP.

β-Catenin encoded by the CTNNB1 gene is an integral cell to cell adhesion adaptor protein as well as transcriptional coregulators. Aberrant action and abnormal expression of β-Catenin have been corroborated in abnormal immune response and various diseases, such as inhibiting TLR4-driven inflammation [21], inducing Treg cells and suppressing TH1/TH17 responses [22], leading abnormal regulation of the Wnt/β-Catenin signaling in MTB infection [12]. There are several lines of evidences investigating the contribution of genetic variant rs4135385 in CTNNB1 gene to tumors risk and prognosis, suggesting its potential function of influencing the development of human diseases. Wang et al. [23] found rs4135385 was significantly associated with an increased risk of gastric cancer and a favorable survival in Chinese population. The significant association between rs4135385 and breast cancer was observed in Alanazi MS' and Lee's research [15], [24]. Our results indicated rs4135385 was correlated with TB, and G allele as well as AG/GG genotype might be risk factors for TB infection. The *p*-value of genotype for rs4135385 was at borderline significant level (0.048), and a larger study will give better indications to the role of this SNP in susceptibility to TB. As rs4135385 is a tag SNP located in the thirteenth intron and close to the fourteenth exon of CTNNB1 gene, its functional difference could be mediated by affecting RNA splicing [25]. Aberrant β-catenin expression and abnormal Wnt/β-catenin signaling could participate in the development and progression of TB. Another possible mechanism is that rs4135385 may be linked with some regulatory SNPs which could change the expression and/or function of β-Catenin, as illustrated in the studies from Wang et al. [26]. However, the exact mechanism underlying the susceptibility of TB conferred by this polymorphism remains to be elucidated.

The Wnt signaling pathway can be blocked by secreted frizzled-related protein 1 (SFRP1), which functions by sequestration of the Wnt ligands and thus avoiding its interaction with its receptor. SFRP1 may act as a novel antiinflammatory factor by switching the balance between proinflammatory and antiinflammatory cytokines [27]. Genetic studies have shown that gene variants in SFRP1 are associated with inflammation and cancer risk [28]–[29]. We explored a polymorphism in SFRP1 (rs78332767) and found significant association with TB risk in Chinese Han population. Individuals with TT genotype were more likely to be infected with TB, indicating SFRP1 may be a candidate gene for tuberculosis determinant. To our knowledge, no data pertaining the analysis of rs7832767 in infectious disease have been reported yet. Further functional studies with ethnically diverse populations are urgently needed. The subgroup analysis identified significant association of this SNP with CRP level, and non-ancestral T allele with elevated CRP value. This not only suggested a clinical significance of this SNP, but indicated its potential to assist in the clinical assessment of the activity and severity of TB. Considering the limited samples, this significance should be confirmed in further clinical studies.

The AXIN2 gene is an integral part of a negative feedback loop that acts to restrain or desensitize canonical Wnt signaling, by helping direct β-catenin for degradation in proteasome [30]–[31]. The mutations and loss of heterozygosity in AXIN2 gene have been identified in various cancers, including lung cancer [32], hepatocellular neoplasm [33], and colorectal carcinomas [34] . Polymorphisms in the AXIN2 gene, including rs11079571, have recently been shown to be associated with increased breast cancer risk [35], suggesting they play a role in cancer susceptibility. Our data revealed a lack of association between rs11079571 and TB risk in Chinese Han population. Although our study indicated an irrelevant result, we believe that our work will improve the understanding of gene variants involved in TB onset and progression.

Several limitations of the present study should be addressed. First, the sample size is not large enough. The associations observed for rs4135385 and rs7832767 with TB need to be validated in larger populations of different ethnicities. Second, our study is only based on a molecular epidemiology survey, and functional analysis of these polymorphisms is warranted in the future studies. Third, other variants, such as SNPs located in the functional region, may also affect susceptibility to TB. Additional genetic analysis within Wnt signaling pathway is required to explore genetic influence factors on the risk of TB.

In conclusion, this is the first survey reporting genetic susceptibility to TB with gene variants of Wnt signaling pathway in Chinese Han population. The results indicated that rs4135385 and rs7832767 were associated with TB susceptibility, and rs7832767 might relate to CRP level in TB patients. Our findings may provide evidence for the role of Wnt signaling pathway as well as screening markers for early detection of tuberculosis. Further large-scale prospective and functional studies are warranted to validate these findings.
